# Deep learning‐based upsampling of 2D detector array measurements for patient plan verification in radiotherapy

**DOI:** 10.1002/mp.70358

**Published:** 2026-03-16

**Authors:** Andreas Pflaum, Nicole Brand, Elias Kempf, Jan Weidner, Daniela Eulenstein, Vanessa Delfs, Björn Poppe, Hui Khee Looe

**Affiliations:** ^1^ University Clinic for Medical Radiation Physics Medical Campus Pius Hospital Carl‐von‐Ossietzky University Oldenburg Germany; ^2^ PTW Freiburg Freiburg Germany

**Keywords:** deep Learning, detector arrays, radiation therapy

## Abstract

**Background:**

Detector arrays are commonly used for treatment plan verifications in intensity modulated radiation therapy. However, the intrinsic resolution of detector arrays is limited by the physical dimensions of each single detector and the detector‐to‐detector distance. This may lead to inaccurate representations of steep gradients and narrow dose peaks.

**Purpose:**

This work presents a deep learning approach for increasing the effective spatial resolution of detector arrays used for patient plan verification. The presented approach aims to augment missing values in the insensitive areas of the detector matrix and to increase the sampling frequency of the measured 2D dose profile. Furthermore, perturbations caused by finite detector's dimensions via the volume‐averaging effect are corrected during the upsampling process.

**Methods:**

In this work, Monte Carlo simulation methods were employed to synthetically generate training data, enabling a wide coverage of different linear accelerator setups and field shapes. The approach was implemented for the OCTAVIUS Detector 1500 (PTW Freiburg, Germany), which consists of 1405 air‐filled ionization chambers arranged in a checkerboard pattern. This arrangement enables a threefold increase in resolution from 5 mm, achieved with the standard bilinear interpolation, to 1.7 mm using neural networks. The implemented neural networks are based on a deep convolutional architecture and were trained using PyTorch. Initially, the models were tested by comparing the upsampled measurements of individual step‐and‐shoot IMRT segments with measurements obtained using a high‐resolution OCTAVIUS Detector 1600 SRS liquid‐filled ionization chamber array. In addition, radiochromic film measurements of fields with leaf gaps of 1 and 2 cm were used to demonstrate the differences between measurements and interpolation results in the presence of steep gradients and narrow dose peaks. Finally, reconstructed 3D dose distributions of VMAT plans, using both the original and upsampled measurements, were compared to the treatment planning system calculations.

**Results:**

The comparison of the individual IMRT segments with a standard bilinear interpolation showed an average increase in the gamma index passing rate of up to 20%. In the case of 3D dose reconstructions from the field‐by‐field IMRT measurements in the OCTAVIUS 4D phantom, the neural network upsampling yielded an average increase in passing rate of 22% as compared to bilinear interpolation, when using the OD 1600SRS array measurements as reference and 19% with the TPS calculated dose distribution as reference. Similarly, for VMAT plans, the passing rate showed an average increase of 8% for the measurement at an Elekta accelerator and 7% at a Varian Ethos accelerator, both using the TPS dose distribution as reference.

**Conclusion:**

It has been shown that a neural network can be applied to upsample the detector array resolution. This results in a better interpolation of measurement points, especially in regions of steep gradients, than compared to a standard bilinear interpolation. The passing rates of all investigated VMAT plans are increased by applying the proposed neural network upsampling approach.

## 1 INTRODUCTION

Verification of treatment plans represents a crucial step in the quality assurance process in modern radiotherapy. State‐of‐the‐art linear accelerators utilize multi‐leaf collimators (MLCs) for complex fluence modulation, enabling the formation of a highly conformal dose distribution that matches the geometry of the target volume while sparing surrounding critical organs.^1^ The resulting treatment plans may comprise large numbers of irregular, small subfields (segments) that are dosimetrically challenging to measure. By using detector arrays with sufficient resolution, treatment plans can be verified. These arrays are characterized by two crucial properties: (1) the detector‐to‐detector distance; and (2) the physical dimensions of the individual detectors.^2^ On the one hand, the detector‐to‐detector distance determines the sampling frequency of the detector array. It has been demonstrated that a sampling frequency of 2.5 mm^−1^ is generally required for a highly‐modulated treatment plan according to the Nyquist theorem.^3^ On the other hand, the finite physical dimensions of the individual detectors and the detector's material will introduce a volume effect. The resulting perturbation can lead to an erroneous dose representation at the point of measurement associated with the corresponding detector. Various methods have been developed to correct for this effect, including the application of correction factors or more generic corrections involving deconvolution techniques.^4^, ^5^, ^6^, ^7^ The interplay between the sampling frequency and volume effect is characterized by the so‐called *coverage factor* as proposed by Stelljes *et al.* ^8^ A detector array with high coverage factor indicates the capability for the array to detect possible dose discrepancies within the array measurement area, that is, the detector's sensitive surface covers a larger portion of the measurement area. For example, the coverage factor of unity implies that the sensitive detector surface covers the complete measurement area.

Nevertheless, constructing detector arrays with a high coverage factor is technically challenging, since this requires a large number of detectors to cover the whole measurement area. Using single detectors with larger volumes would increase the coverage factor, but, as mentioned earlier, this would also imply that the measurements are subject to a more substantial volume effect. Hence, for highly modulated treatment plans, measurements using existing detector arrays may not be sufficient to represent the entire dose distribution, limiting their efficacy and sensitivity in treatment plan verification.

Recently, deep learning approaches have been applied to overcome some of these limitations. Schönfeld *et al.* have demonstrated that the volume effect of ionization chambers associated with 1D profile measurements can be corrected using fully‐connected artificial neural networks.^9^ However, these networks require a large amount of training data, which is typically obtained through measurements. Another challenge is selecting the appropriate detectors to acquire the unperturbed (true) dose profiles for the supervised training process. To overcome these limitations, Looe *et al.* introduced a model‐based training approach by adopting a mathematical convolution model that describes the measurement process to generate artificial training data.^6^ By using Monte Carlo simulated detectors’ lateral response functions, trained neural networks have also demonstrated the ability to recover unperturbed dose profiles in magnetic fields.^6^ Weidner *et al.* also implemented a deep learning approach based on the U‐Net architecture, trained with synthetic data, to correct for the volume effect of the Semiflex 3D chamber (type 31021, PTW Freiburg).^10^ They demonstrated that the results are comparable to those obtained with the microDiamond detector (type 60019, PTW Freiburg). Recently, Looe *et al.* proposed a deep learning framework to predict 2D measurement data from plan data in one direction and vice versa in the reverse direction, thereby allowing detailed segment‐wise plan QA. The models have been pre‐trained using synthetic data and fine‐tuned using limited measurement data.^11^

**FIGURE 1:**
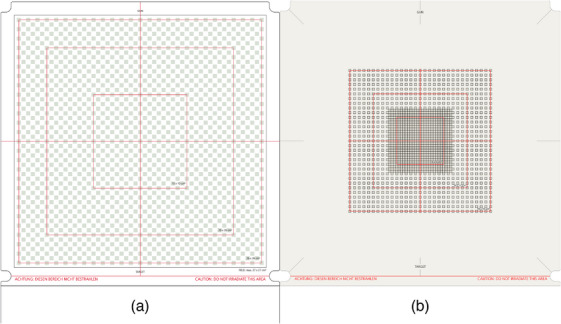
The layout of the ionization chambers in the (a) OCTAVIUS Detector 1500 and (b) OCTAVIUS Detector 1600 SRS.

In this work, the application of deep learning approaches is extended to 2‐dimensional and 3‐dimensional dose measurements. A deep neural network is trained with synthetic data, similar to what was mentioned above, to predict a high‐resolution dose distribution from a lower‐resolution array measurement. The approach was implemented for an ionization chamber array, where pixels are arranged in a checkerboard pattern (see Figure 1a) to increase the sampling frequency by a factor of three. The results were carefully validated and compared to reference measurements acquired with a high‐resolution liquid‐filled ionization chamber array and radiochromic films. Furthermore, the improvements in agreement with the TPS‐calculated dose distributions for clinical VMAT plans were analyzed for 3D dose distributions reconstructed from the upsampled measurements.

## 2 MATERIALS AND METHODS

### 2.1 Detector arrays

In this study, we improve the resolution of the OCTAVIUS Detector (OD) 1500 (PTW Dosimetry, Freiburg)^12^ measurements by applying a deep neural network. The single detectors of the array are arranged in a checkerboard pattern as shown in Figure 1a, with 10 mm and 7.1 mm chamber‐to‐chamber distances, parallel to the main axes and the diagonals, respectively. The sensitive volume of a single chamber is 4.4  × 4.4  × 3.0 mm^3^. A total of 1405 chambers in the array covered a measurement area of 27  × 27 cm^2^.

As a reference, the OCTAVIUS Detector (OD) 1600SRS liquid‐filled chamber array (PTW Dosimetry, Freiburg)^13^ was used due to its higher resolution compared to the OD 1500. The sensitive volume of a single chamber is 2.3  × 2.3  × 0.5 mm^3^ with a sampling distance of 2.5 mm in a 6.5  × 6.5 cm^2^ area (Figure 1b), up to a factor of 4 higher than that of the OD 1500. In the outer area, the sampling distance is 5 mm. A total of 1521 chambers in the array covered a measurement area of 15  × 15 cm^2^.

### 2.2 Upsampling with bilinear interpolation

**FIGURE 2:**
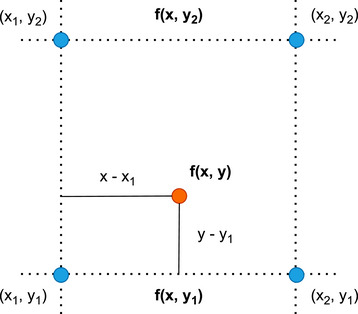
Bilinear interpolation on a rectangular grid.

The results of the deep learning upsampling proposed in this work are compared to those obtained using the standard bilinear interpolation, as the most commonly used technique in dose reconstruction applications. In the implementation of the software (VeriSoft, PTW Dosimetry, Freiburg, Germany) used in this work, the missing values in the checkerboard matrix of the OD 1500 measurements are firstly filled by using the mean of two opposite neighbor values, that is, either along the target‐gun, or left‐right direction (diagonal neighbors are not considered). The subsequent interpolation is then performed by assuming that the value of a function f is given at four points $\left(x_{1},y_{1}\right),\left(x_{1},y_{2}\right),\left(x_{2},y_{1}\right),\left(x_{2},y_{2}\right)$ as illustrated in Figure 2. f(x,y) is given by the following equations

(1) $$f\left(x,y_{1}\right)=\frac{x_{2}-x}{x_{2}-x_{1}}\;f\left(x_{1},y_{1}\right)+\frac{x-x_{1}}{x_{2}-x_{1}}f\left(x_{2},y_{1}\right)$$

(2) $$f\left(x,y\right)=\frac{y_{2}-y}{y_{2}-y_{1}}\;f\left(x,y_{1}\right)+\frac{y-y_{1}}{y_{2}-y_{1}}f\left(x,y_{2}\right)$$

for $x_{1}\leq x\leq x_{2}$ and $y_{1}\leq y\leq y_{2}$.

### 2.3 Generation of training data

**FIGURE 3:**
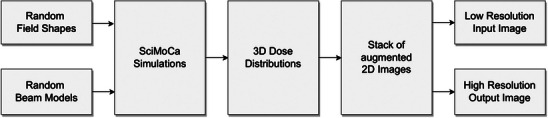
Schematic illustration of the data generation process.

The data used to train the neural network is entirely generated synthetically. The algorithm has been implemented to cover all possible clinical scenarios, ensuring the robustness of the trained model. Figure 3 shows a high‐level overview of the algorithm. In the following, the general principles of the algorithm are outlined.

For the supervised training, pairs of low‐resolution and high‐resolution measurements are required. The former represents the measurement data obtained with the OD 1500 (input), which consists of 53 × 53 pixels, each of size 5 mm × 5 mm. The latter is the output of the neural network with an effective resolution of 159 × 159 pixels and a pixel size of 1.67 mm × 1.67 mm. The neural network was designed to accept only OD 1500 measurements as input and to operate independently of other potentially relevant features, for instance, the nominal beam energy and measurement depth. By randomly sampling these features in the training data, they can be implicitly considered by the neural network. Therefore, the training data should be comprehensive so that the trained neural network can operate correctly under all clinical measurement conditions. For example, the detector could be placed in an OCTAVIUS 4D phantom (PTW Dosimetry, Freiburg), an OCTAVIUS II phantom (PTW Dosimetry, Freiburg), or under a stack of RW3 slabs. Additionally, the same neural network can be used for all clinically relevant photon beam qualities, linear accelerator models, source detector distances, collimator angles, field shapes, and so on. These objectives and constraints guided the design of the data generation algorithm and have been chosen according to the parameters presented in Table 1. The dose distributions obtained from the SciMoCa data generation process are given in Gy based on TG‐51, so that no dose conversion is required.

**TABLE 1:** Parameters and constraints for the data generation algorithm.

Field shapes	67% spot openings generated by sampling in‐field points and fitting the MLC leaves to them 22% rectangular fields 11% random MLC patterns Maximum field size 330 mm x 330 mm
Gantry angle	20% at 0° 80% uniform sampling from −18° to 18°
Collimator angle	20% uniform sampling in the range [−180°, 180°] 20% at 0° 20% discrete sampling at −90°, 90° and 180° 20% discrete sampling at −135°, −45°, 45° and 135° 20% normal distribution (*σ* = 11.25°) around discrete sample of −135°, −90°, −45°, 0°, 45°, 90°, 135° and 180°
Couch angle	90% at 0° 10% same procedure as gantry angle
MU count	Uniform sampling from 50 MU to 150 MU
Beam Model	Nominal energy: sampled from generalized extreme value distribution with (mu = 7 MeV, sigma = 6 MeV, eta = 0.1 MeV) truncated to^14^, ^15^ MeV
Source‐to‐surface distance	80 cm
Source‐to‐detector distance	80–120 cm (in 1.67 mm steps depending on slice depth)
MLC model	Elekta Agility

**FIGURE 4:**
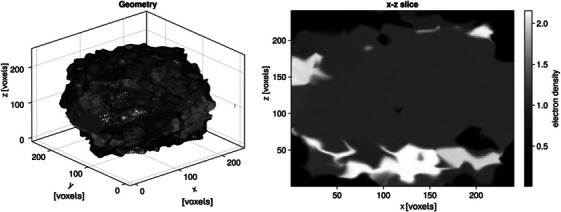
Example of a randomly generated geometry and a corresponding slice through the *x*–*z* plane, i.e., the detector plane used for the dose calculations.

The resulting algorithm can be subdivided into three phases: 1. the random generation of simulation parameters, 2. a Monte Carlo simulation of dose distribution for each parameter set, and 3. the extraction of data points from the simulated dose distributions:

1. In the first phase, the parameters needed to perform a full Monte Carlo dose simulation are sampled randomly. These parameters include the photon energy, and the field shape. To cover an extensive and sufficient variety of clinically relevant field shapes, a mixture of the following field generation strategies was used: (i) random generation of rectangular fields, (ii) random positions of each MLC leaf and jaws with only restrictions of the maximum field size, and (iii) randomly sampled points in the MLC plane to create the smallest field shape that contains every sampled point. This procedure ensures that, while covering the entire clinically relevant parameter ranges, the generated data is not biased towards the characteristics of a specific series of linear accelerators.
2. For the combinations of selected parameters in (1), 3D dose simulations were performed using SciMoCa, a Monte Carlo algorithm commonly used for secondary dose checks and plan QA (ScientificRT, Germany). To ensure the trained neural network works with different clinical build‐up geometries (array could be placed in an OCTAVIUS 4D phantom, an OCTAVIUS II phantom, or under a stack of RW3 slabs), the Monte Carlo simulations were performed with varying inhomogeneous voxelized phantom geometries with a resolution of 1.67 mm x 1.67 mm x 1.67 mm. A random process was used to procedurally create these inhomogeneous voxelized geometries with electron densities between 0.01 and 20 times the electron density of water. This step further introduced randomization in the form and shape of the profiles, including the penumbra regions to ensure high generalization of the resulted dose distributions. Figure 4 presents a randomly generated geometry together with a corresponding cross‐section through the *x*–*z* plane at a voxel count of *y* = 120. It should be noted that, in SciMoCa, the *y*‐axis denotes depth; therefore, the detector array is oriented parallel to the *x*–*z* plane.
3. The training pairs consist of a down‐sampled simulated dose distribution representing the detector measurement and the corresponding high‐resolution simulated dose distribution. For this purpose, each 3D dose cube is sampled as a stack of 2D dose slices along the beam's axis. Firstly, the simulated high‐resolution dose distribution is sampled randomly as a sub‐matrix within one 2D dose slice. Additionally, data augmentations, for example, a rotation by a multiple of 90° or reflection along one of the symmetry axes of a square, were applied to the submatrix. This combination of subsampling and data augmentation drastically reduces the correlation between slices originating from the same dose cube. The corresponding down‐sampled dose maps, which later serve as input for the neural network, are obtained by average pooling the high‐resolution dose distributions with a kernel size of three‐by‐three. A Gaussian noise is added to all the down‐sampled dose maps, where the pixels not covered by an ionization chamber are zeroed out. As a result of the implemented sampling process, the neural network will also account for the correction of volume‐averaging effect caused by the physical dimensions of the chamber's sensitive volume.

**FIGURE 5:**
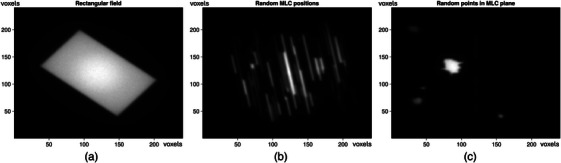
Examples of the training data. (a) a rectangular field, (b) random MLC positions, (c) random points in MLC plane.

Following the procedure described above, a data set containing around 300 000 data pairs from 1280 dose simulations was created. The dataset was split into training and validation samples, with the validation set comprising approximately 15% of the total samples. Note that this subdivision was performed on the underlying simulation data rather than the extracted training pairs. This ensures that data derived from the same simulation, which show correlation to each other, are not split between training and validation data. This allows the reliable detection of model overfitting during the training. Figure 5 shows examples of a rectangular field (a), random MLC positions (b) and random points in the MLC plane (c) contained in the training data.

### 2.4 Structure and training of the neural network

**FIGURE 6:**
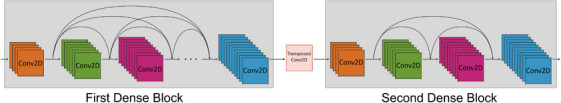
Structure of the proposed neural network.

A variation of DenseNet was chosen as the architecture of the neural network.^16^ The high‐level structure of the network is shown in Figure 6. Each dense block consists of densely connected convolution layers, that is, each layer can access the outputs of all previous layers within the same block. The first dense block consists of 16 layers, and the second block consists of 4 layers. Both dense blocks use a growth rate of 8. In between the two dense blocks is a transposed convolution layer using a 3 × 3 kernel and a stride of 3. The growth rate defines the increase in the number of feature maps (channels) between two consecutive layers within a dense block. For example, starting with 16 feature maps and a growth rate of 8 results in successive layers with 16 → 24 → 32 feature maps. Thus, the growth rate controls the ‘width’ of the network, in contrast to its ‘depth,’ which is determined by the number of layers.

The neural network was implemented in PyTorch, an open‐source machine learning framework.^17^ The neural network was trained for 10 days on a NVIDIA Tesla V100 PCIe GPU. The training hyperparameters used for the neural network are shown in Table 2.

**TABLE 2:** Hyperparameters for the training of the neural network.

Training hyperparameters	
Epochs	112
Batch Size	32
Initial Learning Rate	1e−3
Learning Rate Schedule	Cosine annealing with warm restarts^18^ (restarts at epochs 16 and 48)
Optimizer	AdamW^19^
Loss Function	MSE + Equivariance Loss

**FIGURE 7:**
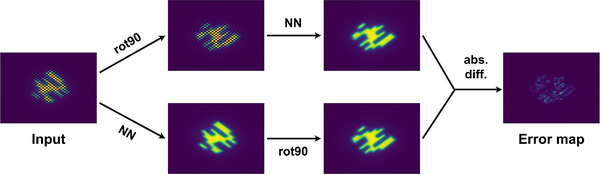
Computation of equivariance loss. For a given input and a given transformation (e.g., rotating by 90°), the equivariance error map is computed by comparing the prediction for the transformed input to the transformed prediction for the untransformed input. The final loss is obtained by applying MSE to the error map.

The loss function used to train the neural network consists of two components. The first component consists of a basic mean‐squared error (MSE) between the predicted high resolution dose distribution—obtained as output of the neural network from the down‐sampled simulation (input)—and the original high‐resolution dose distribution, both with an effective resolution of 159 × 159 pixels and a pixel size of 1.67 mm × 1.67 mm. The second component is an equivariance error,^20^ which enforces consistency with certain transformations, i.e., rotations and reflections. Figure 7 shows an example of the computation of the equivariance error on the output of the neural network. In addition to the output, the equivariance error is also computed at all intermediate dense layers. Finally, the MSE and the equivariance errors are summed up, and the compound loss function is then used to train the neural network.

### 2.5 Measurements of test data

#### 2.5.1 Step‐and‐shoot IMRT segments

The trained deep learning approach was tested using a total of 159 segments measured at an Elekta Synergy linear accelerator, as well as 164 segments measured at a Varian TrueBeam linear accelerator with a 6 MV photon beam, extracted from eight clinical step‐and‐shoot intensity‐modulated radiation therapy (IMRT) fields. Every IMRT segment was measured individually with the OD 1500^12^ and OD 1600 SRS,^13^ both embedded in a 30 cm × 30 cm RW3 slab phantom with 10 cm RW3 above and beneath the detector arrays, at a source‐to‐surface‐distance (SSD) of 90 cm.

The arrays were cross‐calibrated under the same measurement conditions as described above against absorbed dose‐to‐water at the point of measurements of the central chamber. The dose was measured with a Semiflex 31010 chamber (PTW Freiburg) following the German DIN 6800–2 protocol. For the OD 1500, a field size of 10 cm × 10 cm was used. A smaller field size of 4 cm x 4 cm was used for the cross calibration of the OD 1600 SRS.

The agreement between the output of the neural network and the OD 1600 SRS measurements was evaluated based on the gamma index passing rate. The gamma index computation was performed in VeriSoft. The gamma passing rate analysis was carried out by applying a 2%/2 mm local dose criterion for all IMRT segments with 10% dose threshold suppression.

#### 2.5.2 Fields with leaf gaps

Besides the IMRT segments, manually generated fields with different gap widths modulated by closing and opening subsequent MLC leaves were measured using the arrays and with EBT3 films. The calibration of the films has been performed with 10 dose points between 0 Gy and 2 Gy. Each calibration point was repeated three times. Each piece of film was scanned separately at a resolution of 72 dpi and 48 bit (16 bit for each color channel). For the calibration and evaluation of the measurements, only the red channel was used. The pixel values *px* of the scanned films were converted to mean transmittance *T* according to equation (3).^21^

(3) $$T=\log_{10}\left(px*2^{16}\right)$$

The calibration curve is fitted using a third order polynomial

(4) $$D\left(T\right)=p_{1}*T^{3}+p_{2}*T^{2}+p_{3}*T+p_{4}$$

The fit parameters are denoted as *p*_1_ to *p*_4_.^22^

### 2.6 Gamma index analysis

#### 2.6.1 IMRT and VMAT plan verifications

The 3D dose distributions of eight IMRT fields reconstructed from field‐by‐field measurements in an OCTAVIUS 4D phantom using the OD 1500, resulting from bilinear interpolations and neural network upsampling, were compared.

Additionally, a similar comparison was also performed using 3D dose distributions of VMAT plans. In this work, a set of 26 VMAT plans for an Elekta Synergy linear accelerator, consisting of 8 prostate, 9 head‐and‐neck, and 9 lung cases, and another set of 18 plans for a Varian Ethos accelerator, were analyzed.

For both the field‐by‐field IMRT plans and the VMAT measurements, the Gamma index criteria were set to 2 mm and 2% with 20% dose threshold suppression. The reconstruction voxel size was set to 1.7 mm side length to match the grid size of the neural network upsampled dose distributions.

## 3 RESULTS

### 3.1 Training

**FIGURE 8:**
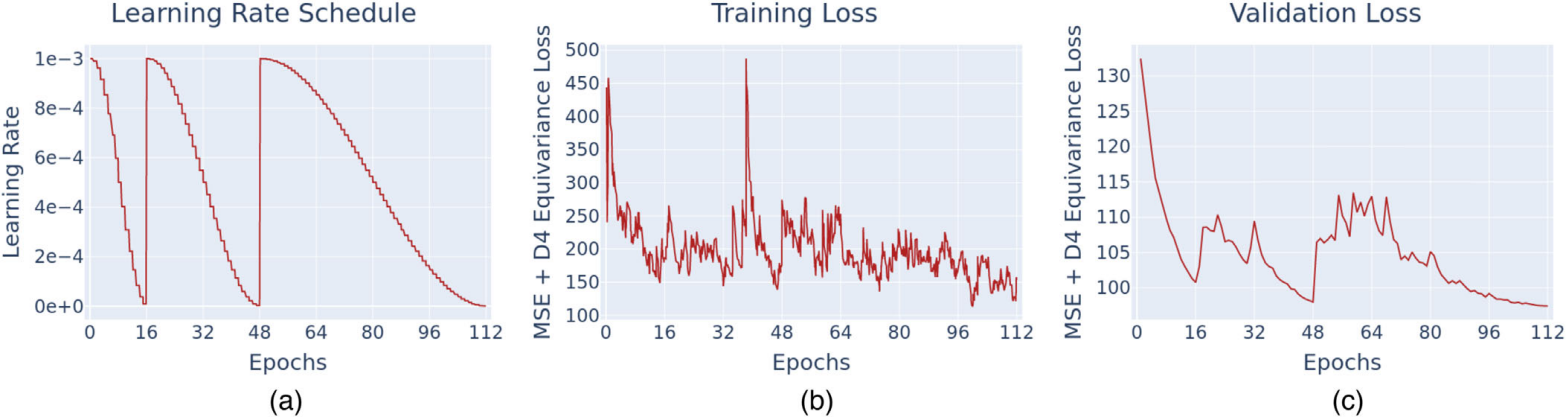
Training metrics recorded during the training process: (a) the learning rate schedule used, (b) loss on the training set, and (c) loss on the validation set.

Figure 8 shows the results of the training process with a cycle‐based learning rate schedule (Figure 8a). Both training and validation losses (Figure 8b and 8c respectively) show a decreasing tendency within each learning rate cycle. One can also observe that the validation loss does not decrease as strongly during the third learning cycle (between epochs 48 and 112) compared to the first two cycles, that is, after 112 epochs, it is only marginally lower than after 48 epochs. This suggests that the neural network had nearly converged, and further training would not yield substantial performance improvements.

### 3.2 Test data

#### 3.2.1 Step‐and‐Shoot IMRT segments

**FIGURE 9:**
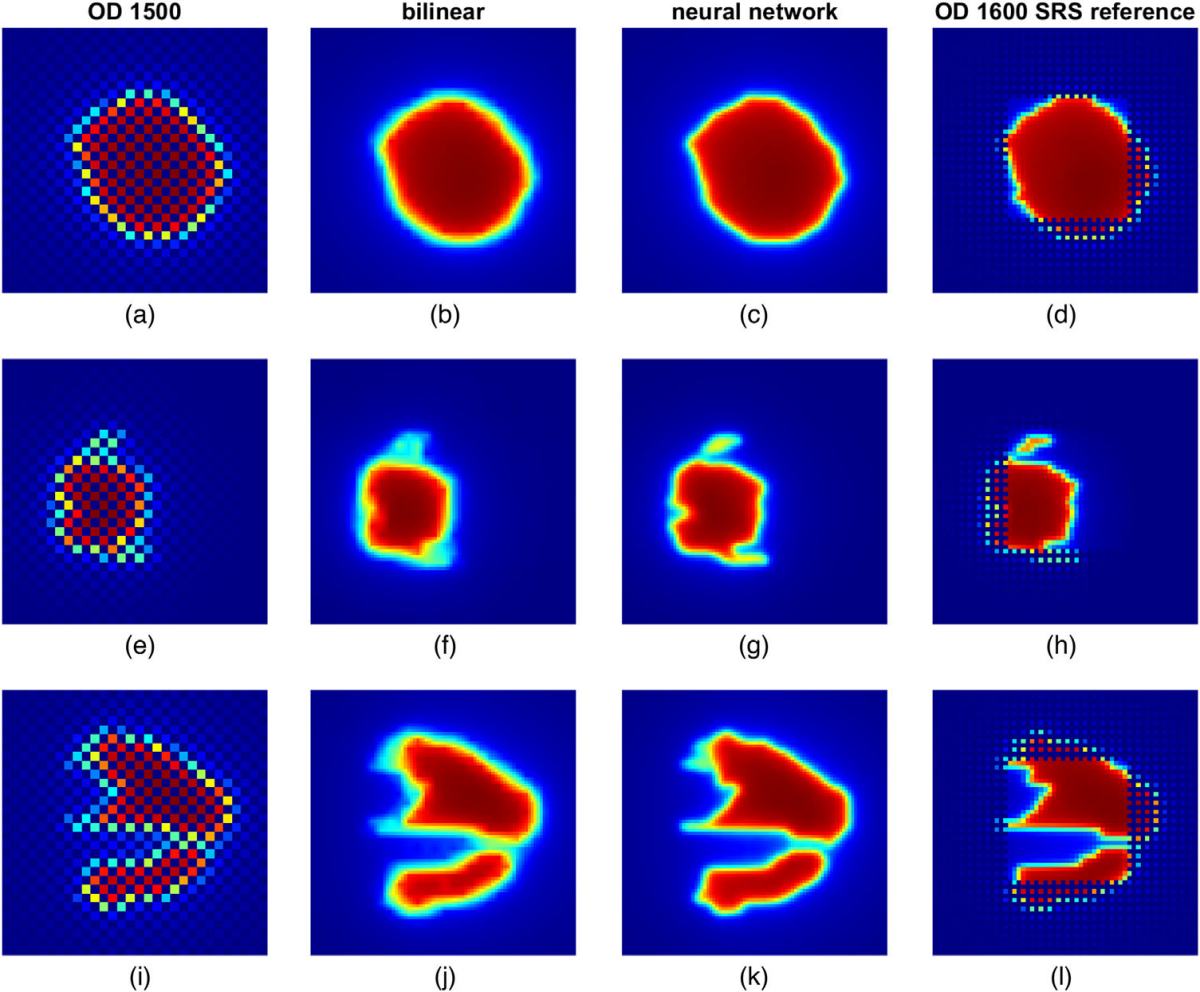
Comparisons between the OD 1500 measurements, high‐resolution measurement obtained using bilinear interpolation, high‐resolution measurements predicted by trained neural network and high‐resolution measurement obtained using OD 1600 SRS for three IMRT segments (segment 1: subfigure (a–d), segment 2: subfigure (e–h), segment 3: subfigure (i–l)).

Figure 9 shows an example of the comparisons between the OD 1500 measurements (Figure 9a,e and i), high‐resolution measurements obtained using bilinear interpolation (Figure 9b,f and j), predicted high‐resolution measurements obtained using the trained neural network (Figure 9c,g and k), and high‐resolution measurements obtained using OD 1600 SRS (Figure 9d,h and l), for three IMRT segments. Compared to bilinear interpolation, it can be visually perceived that the neural network predicted high‐resolution measurements reproduce more fine details of the underlying dose distributions.

**FIGURE 10:**
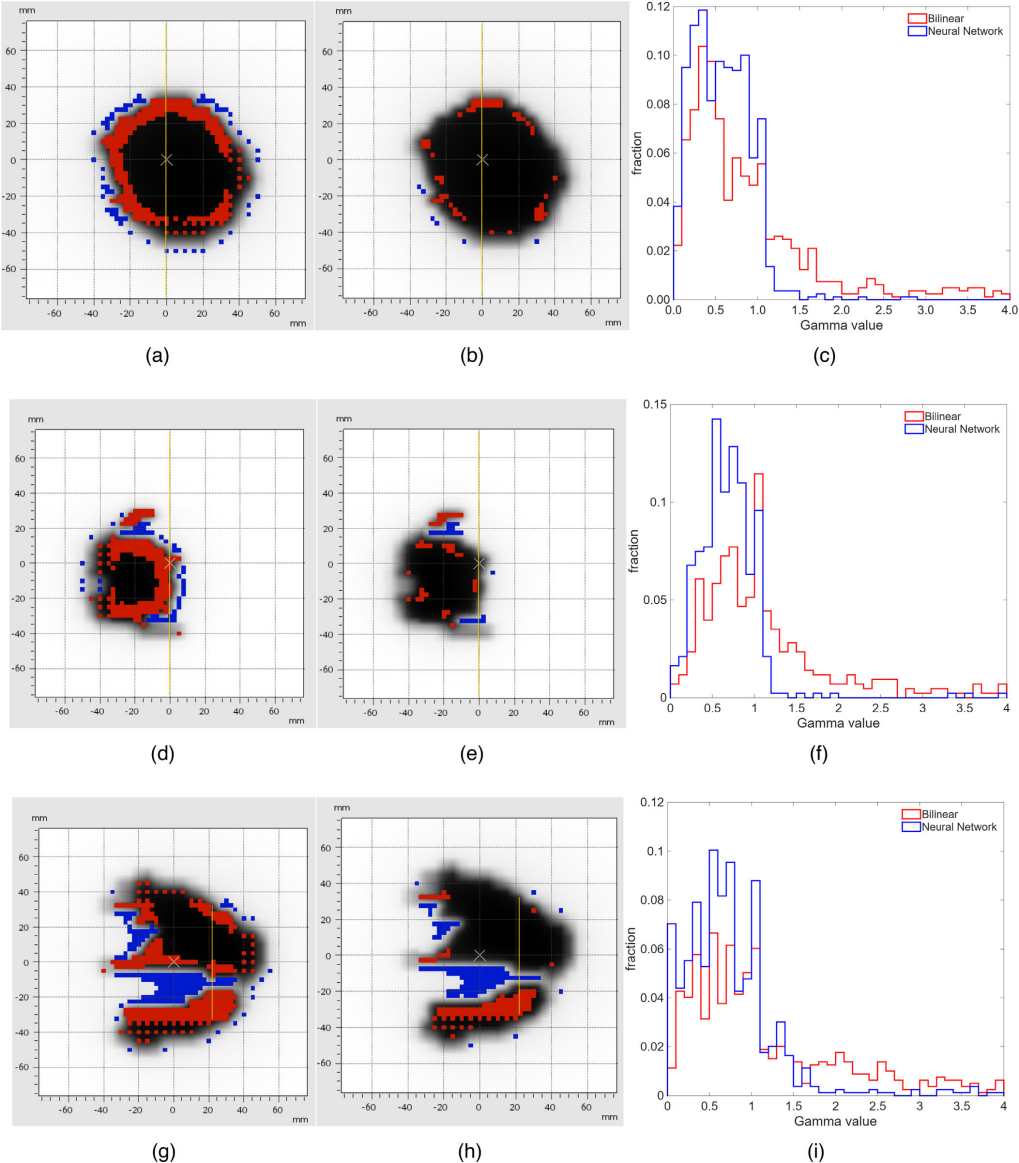
The results of gamma index analysis for the three IMRT segments presented in Figure 9 between the high‐resolution measurements obtained using bilinear interpolation (subfigures a, d and g) and neural network (subfigures b, e and h) by using the OD 1600 SRS measurements as reference. The failed points in red and blue color are marked in the figure. Subfigures c, f and I show a histogram for each segment truncated at Gamma = 4 to illustrate the magnitude in Gamma value of the failed points.

By using the OD 1600 SRS as the reference, the gamma passing rates for the bilinear interpolation were found to be 65.8%, 50.7% and 47.6% for the segments in rows 1, 2 and 3, respectively, whereas the passing rates increased to 92.6%, 86.4%, and 73.5 % for the corresponding segments when using the neural network predictions. In all three segments, the majority of these failed points were found in regions with steep and strongly varying dose gradients as indicated in Figure 10. Histograms of the gamma values for each segment show a shift toward lower values for the neural network predictions compared to the bilinear interpolation, reflecting improved dose agreement. The histograms have been truncated at a gamma value of 4.

**FIGURE 11:**
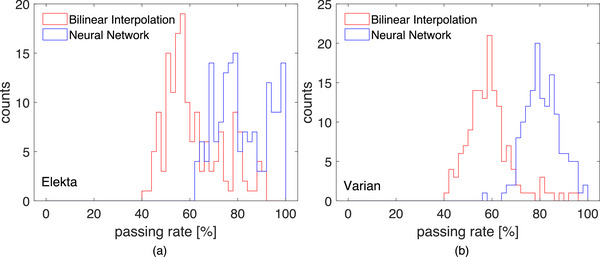
Histogram of the gamma passing rates of bilinear interpolation and the neural network predictions for all investigated IMRT segments, (a) for the Elekta Synergy plans, (b) for the Varian trueBeam plans.

The gamma passing rates of all 159 evaluated IMRT segments are presented in Figure 11. In the case of the Elekta accelerator, the mean passing rate for the bilinear interpolation was (61.8 ± 12.2)%, while the mean passing rate for the neural network predictions is (81.7 ± 10.9)%. The Varian data showed a passing rate of (58.8 ± 9.6)% for the bilinear interpolation and (81.0 ± 7.3)% for the neural network prediction.

#### 3.2.2 Modulated fields with leaf gaps

**FIGURE 12:**
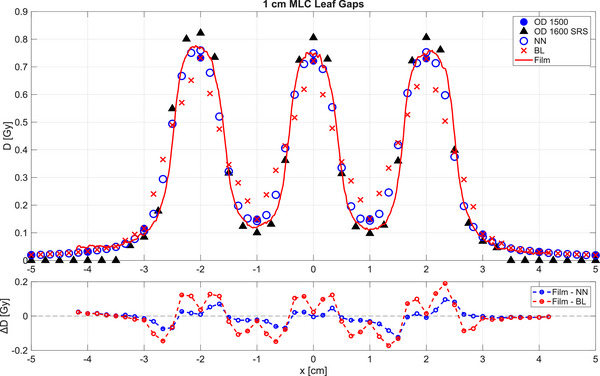
1D profiles of the field with 1 cm leaf gaps measured by the OD 1500, upsampled with bilinear interpolation (BL), predicted by neural network (NN), measured with OD 1600 SRS and EBT3 films. Additionally, a plot for the difference between film measurement and BL as well as NN, ∆D in Gy, has been included.

To better understand the differences of gamma passing rates between bilinear and the proposed neural network interpolations, modulated fields were measured with EBT3 films, the OD 1500 and the OD 1600SRS arrays. The interpolated OD 1500 measurements are plotted alongside the original OD 1500, the OD 1600SRS and the film measurements in Figure 12 for a field with 1 cm leaf gaps as an example. The profile measured using the OD 1600SRS shows good agreement with the film measurement. Nevertheless, a small over‐response and under‐response of the OD 1600 SRS measurement can be observed at the peaks and valleys, respectively. This effect is attributed to the density perturbation of the array caused by the presence of enhanced‐density materials in the detector's housing and has been previously described in the literature for single detectors.^15^, ^23^, ^24^

A difference plot has been included in Figure 12 to illustrate the deviations between the film measurement and the two interpolation methods, showing that the neural network output aligns more closely to the film measurements than the standard bilinear interpolation. The uncertainty associated with the film dosimetry and calibration was small compared to the observed dose differences between the film measurements and the two reconstruction approaches and therefore does not affect the interpretation of the results shown in Figure 12.

The OD 1500 measurement points with 10 mm distance are sparsely distributed along the profile so that the dose modulation cannot be sufficiently captured. On the one hand, the application of simple bilinear interpolation to up‐sample the OD 1500 measurement introduced erroneous points between the real measurement points that deteriorate the gamma passing rate, as has been demonstrated in Figure 11a and b. On the other hand, the predicted high‐resolution measurement using the trained neural network in this work reproduces to a high degree of accuracy the steep and strongly varying dose gradient in this highly modulated field, with good agreement to both the film and OD 1600 SRS measurements. These observations are reflected in the gamma analysis, where the gamma passing rates of this 1 cm gap field amount to 78.7% and 97.7% for both the bilinear interpolation and neural network prediction, respectively. For the field with a 2 cm leaf gap (results not shown here), the gamma passing rates of the bilinear and neural network interpolations are 80.7% and 91.3%, respectively.

### 3.3 IMRT and VMAT plan verification

#### 3.3.1 IMRT

The bilinear interpolated and the deep learning upsampled field‐by‐field IMRT measurements of the OD 1500 array were compared to either the OD 1600 SRS measurements or the TPS calculations as reference. The passing rates of the deep learning based upsampling method shows better agreement to both the OD 1600 SRS measurements and TPS, where the passing rates are improved by up to 30 percentage points as shown in Table 3.

**TABLE 3:** Results of a gamma index analysis for the original and deep learning upsampled OD 1500 measurements of eight IMRT fields on a field‐by‐field basis using either the OD 1600 SRS measurement or the TPS as reference. The passing rates of the neural network output are up to 30 percentage points higher.

Field	OD 1500 bilinear interpolated (Ref.: 1600SRS)	Neural network (Ref.: 1600SRS)	OD 1500 bilinear interpolated (compared to TPS)	Neural network (compared to TPS)
Brain 1	52.4%	84.2%	71.3%	92.2%
Brain 2	77.0%	96.2%	83.6%	98.3%
Brain 3	65.4%	91.2%	69.0%	85.7%
Brain 4	67.9%	95.8%	78.3%	95.4%
Brain A	74.3%	94.4%	73.0%	93.2%
Brain B	69.4%	93.7%	70.6%	88.2%
Brain C	80.2%	97.5%	80.1%	95.7%
Brain D	82.0%	98.4%	85.6%	95.4%
**Total (8 fields)**	**(71.2 ± 8.9)%**	**(93.9 ± 4.2)%**	**(74.4 ± 9.8)%**	**(93.0 ± 3.9)%**

#### 3.3.2 VMAT

The results of the VMAT measurements show a consistent improvement in passing rates across all plan types when using the neural network upsampled measurements compared to the bilinear interpolated OD 1500 data as presented in Table 4.

**TABLE 4:** Mean passing rates for 8 prostate, 9 head and neck, as well as 9 lung plans for a total of 26 plans with a gamma criterion of 2 mm 2%. The table shows improvements for the neural network (NN) over the bilinear (BL) interpolated OD 1500 measurements.

2 mm 2%	TPS vs. BL	TPS vs. NN
Prostate (8 plans)	(83.2 ± 4.1)%	(93.1 ± 2.6)%
Head and Neck (9 plans)	(83.6 ± 3.5)%	(90.5 ± 2.8)%
Lung (9 plans)	(86.6 ± 5.4)%	(94.1 ± 2.9)%
**Total (26 plans)**	**(84.5 ± 4.8)%**	**(92.6 ± 3.2)%**

The same procedure has been applied to 18 VMAT plans of various entities measured at a Varian Ethos accelerator. The results here showed a passing rate of (86.3 ± 3.8)% for the bilinear interpolated dose distributions and (93.7 ± 2.1)% passing rate for the neural network output.

**FIGURE 13:**
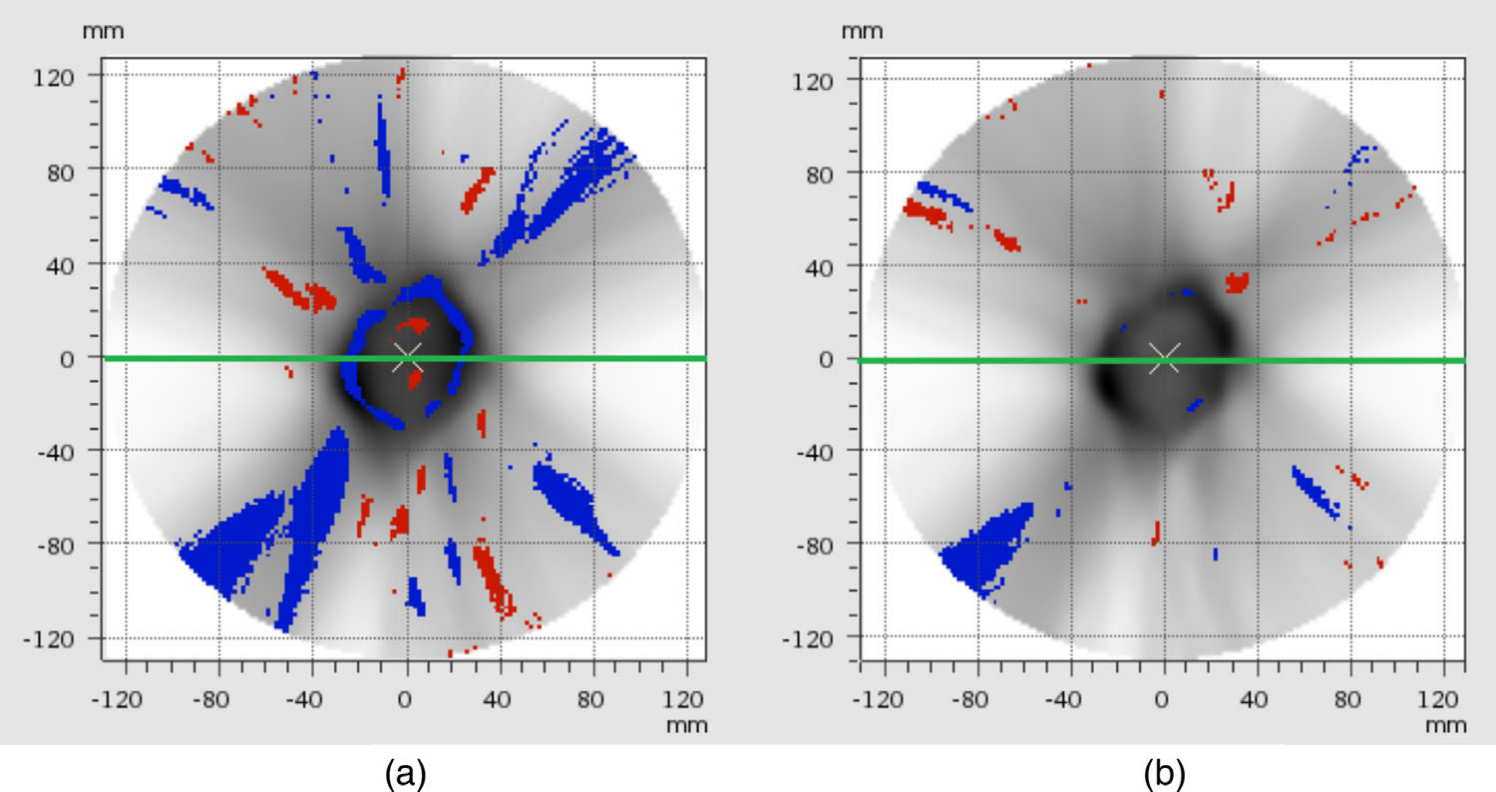
Example of a cross section through a cylindric VMAT dose volume. Left: Failed points map of the standard bilinear interpolation when comparing to the TPS calculated dose distribution. Right: Comparison of the neural network output and the TPS calculated dose distribution.

Figure 13 shows that the number of failed points decreases in a cross‐section plane through the isocenter when the neural network is applied (right) compared to the standard bilinear interpolation (left).

**FIGURE 14:**
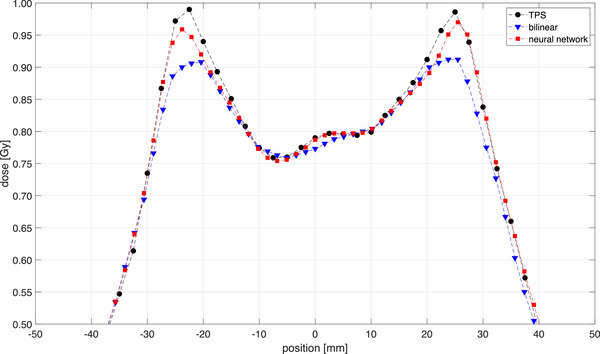
Line profiles through the dose plane shown in Figure 13. The green line in Figure 13 indicates the position of the line plot. The dose profile shows the standard bilinear interpolation (blue) and neural network interpolation (red) compared to the TPS calculated dose profile (black).

An example of a line plot through the same dose plane as in Figure 13 shows that the neural network approximation of the dose peaks (red markers) is closer to the planned dose calculated by the TPS (black markers) than in the standard case (blue markers). The plot is shown in Figure 14.

## 4 DISCUSSION

One of the most crucial components of deep learning systems is the availability of high‐quality training data. When solving image regression tasks as per the aims in this work, neural networks require a large number of data samples to achieve reliable performance and proper generalization. Creating such large data sets from real, high‐quality measurements is very challenging, especially when the data collected must cover a broad range of clinically relevant setups, such as different linear accelerators, beam energies, and field shapes. Therefore, the development of algorithms dedicated to generating high‐quality synthetic data represents an essential step in maximizing the potential of deep learning models in this domain. Nevertheless, it is equally important to assess the potential and limitations of neural networks trained on synthetic data. One aim of this work is to demonstrate that neural networks trained on carefully designed synthetic data can generalize well to real measurement data encountered in clinical practice. This has been achieved by exploiting the flexibility of a state‐of‐the‐art Monte Carlo dose calculation algorithm to generate high‐quality, realistic clinical dose distributions for a large number of randomly sampled irradiation conditions, including beam energy, collimator distances, and field shape.

The task to be solved exhibits a high degree of locality, that is, the predicted value of a missing pixel is heavily influenced by close‐by pixels or direct neighbors, but should depend only slightly or not at all on pixels far away. These kinds of tasks are most appropriately modeled by convolutional neural networks (CNNs). Although most modern CNN architectures are expected to achieve reasonable results, using more sophisticated architectures would improve data efficiency (i.e., reducing the amount of training samples needed) and gradient flow. Thus, a variation of DenseNet by Huang et al.^16^ was chosen as the base structure of the neural network in this work.

The neural network presented in this work has been trained to increase the sampling frequency by a factor of three, that is, the pixel size is decreased from 5 mm x 5 mm to 1.67 mm x 1.67 mm. The final resolution of the upsampling process has been chosen carefully, taking into consideration the required sampling frequency to reconstruct all clinically relevant details of the underlying dose distributions from the measurements, as well as the computational efficiency for training and inference of the neural network. In earlier works, it has been demonstrated that in clinical dose distributions of highly modulated IMRT plans, spatial frequencies of up to 0.1 mm^−1^ are encountered.^2^, ^3^ Therefore, a minimum sampling frequency of 0.2 mm^−1^ should be reached according to the Nyquist theorem. With a target resolution of 159 × 159 pixels of size 1.67 mm x 1.67 mm each, the presented approach reaches a sampling frequency of 0.6 mm^−1^. Further resolution increments would come at much higher computational cost for generating the training data and for training the neural network. Nevertheless, the effort is also expected to yield diminishing returns as it exceeds the highest clinical requirements. Consequently, the chosen target resolution represents a good balance between achieving sufficiently high‐resolution measurements, even for highly modulated dose distributions, and reasonable computational costs.

The Monte Carlo dose distributions used for training were generated using SciMoCa with predefined statistical uncertainty levels. As inherent to Monte Carlo methods, the dose values are associated with statistical uncertainties, which were controlled by selecting a nominal minimum uncertainty that balances computational effort and statistical stability. In this work, the “Extra Fine” setting was used, corresponding to a nominal minimum statistical uncertainty of approximately 0.5% in the high‐dose region. The chosen uncertainty levels were sufficiently low to ensure physically plausible and smooth dose distributions suitable for network training. It took 4 days to generate the training data in this work with 2 x Intel Xeon Platinum 8272CL processors (32 cores total, 64 threads total). Further details on the Monte Carlo dose calculation framework and uncertainty concept are described in the SciMoCa documentation (Scientific RT, Munich, Germany) as well as in the literature by Hoffmann *et al.* ^25^ The training took 10 days on a NVIDIA Tesla V100 PCIe GPU. The requirements of the clinical user to run such a model are very low, with an inference time of only approximately 10 ms per segment, where a complete treatment plan would take about 1–2 s.

Monte Carlo data generation was performed using the Elekta Agility model in this work. Considering that one of the aims of this work is to produce linac‐independent models, we took careful steps to ensure high generalization of the trained models, including broad randomization of beam parameters such as source sizes, energies, field shapes, as well as dose modulation with an inhomogeneity phantom. This has been tested in this work using measurements acquired at a Varian accelerator.

First, it had to be demonstrated that the neural network upsampling yielded a dose distribution that corresponded to the true dose distributions for individual segments. For this purpose, the true dose distribution has been measured with films for leaf gap fields with 1 and 2 cm gap widths and compared to the OD 1600SRS measurements of the same fields, which offer a higher spatial resolution than the OD 1500 measurements. The films and the OD 1600SRS measurements were in good agreement. The latter have therefore been chosen as reference representing the true dose distribution, since the advantage in time efficiency of array measurements has been a key factor.

The neural network trained using synthetically generated training data has been tested using independent data acquired through measurements of single segments of step‐and‐shoot IMRT plans. The outputs of the neural network have been compared to the measurements of the OD 1600 SRS as the reference. For comparison, the measurements were upsampled using bilinear interpolation as performed in most dose reconstruction algorithms. The gamma index analysis has shown that the output from the neural network resulted in significantly higher passing rates than bilinear interpolation (mean 81.7% for the neural network vs 61.8% for the bilinear interpolation for the Elekta measurements and 81.0% for the neural network vs 58.8% for the bilinear interpolation in the case of the Varian Truebeam measurements). The increase in gamma passing rates observed for VMAT plans is less pronounced than for individual step‐and‐shoot IMRT segments. This can be attributed to the composite nature of VMAT dose distributions, which typically results in large regions of relatively homogeneous dose, while the fraction of detectors exposed to steep dose gradients is reduced compared to single‐segment measurements. Visual comparisons between the dose distributions presented in Figure 9 and 10 indicate that the neural network output can better represent the regions of steep and strongly varying dose gradients. This is in line with the results published in the work of Schönfeld *et al.*,^9^ where it was shown that convolutional neural networks can return a better approximation of 1D‐profiles in small fields by correcting the volume averaging effect of ionization chamber measurements, which is also the case for the neural network discussed in this paper. However, the approach presented in this work shows that the neural network is also applicable to more complex dose distributions. Consequently, the failed points in the gamma index analysis in these regions are reduced.

Additionally, the comparison shown in Figure 12 of an MLC field with 1 cm gaps demonstrated that the output of the neural network agrees with the film measurement. In contrast, the bilinear interpolation introduces erroneous points between the actual measurement positions. It is noteworthy that the measurement of the OD 1600 SRS used as the reference in this work also shows good agreement to the film measurement. Nevertheless, it is also evident that the OD 1600 SRS measurement over‐responds at the dose peaks and under‐responds at the dose valleys. This behavior of the OD 1600 SRS is attributable to density perturbations.^26^

In the final step, the impact of the deep learning upsampling on the reconstruction of the 3D dose distribution of VMAT plans has been investigated. An improvement of about 8% points on average in passing rates for a gamma criterion of 2 mm/2% has been shown.

The observed increase in passing rates by applying the proposed neural network approach can be attributed to better approximation in regions of high dose gradients and in small fields, which are more prone to errors attributed to the detector's volume effect. This translates to a higher number of dose voxels failing the gamma index test when the standard bilinear interpolation is used.

The reported results suggest that the neural network approach is capable of partially recovering information lost due to the aforementioned averaging effects introduced by the OCTAVIUS Detector 1500 and therefore offering a more reliable approximation of the true dose distribution. The clinical benefit lies in the more detailed reconstruction of dose distributions, especially in fields with steep gradients, which is often the case in highly modulated treatment plans and in stereotactic radiotherapy, where sparing normal tissue surrounding the target volume is most crucial. In future work, this approach can also be extended to OD 1600SRS measurements to increase the resolution of stereotactic treatment plan verifications, which rely on as accurate dose verification measurements as possible. Since time consuming MC methods require a high amount of computational power and resources, the proposed deep learning model offers an efficient method for fast and practical calculation of upsampled dose distributions in clinical application and can be included in daily treatment plan verification.

Using the same strategy as presented in this work, the models can be adapted using specific training data tailored to various measurement devices with different configurations, such as dimensions, sampling distance and various incidence angles.

## 5 CONCLUSION

In this work, a neural network has been trained to upsample the resolution of the OD 1500 measurements, achieving a sampling distance of 1.67 mm. Furthermore, the output also accounted for the volume‐averaging effect caused by the physical dimensions of each chamber. The predicted high‐resolution measurements show better agreement with both the OD 1600 SRS and film measurements, as compared to conventional bilinear interpolation. The presented approach supports machine and patient QA, which are the common tasks performed with an OD 1500 and is generalizable to all measurements with an OD 1500. The higher resolution is expected to increase the accuracy of 3D dose reconstruction, for example, when used with the OCTAVIUS 4D phantom, especially for highly modulated treatment plans with steep and varying dose gradients. Due to the promising results for the OD 1500 presented here and the need for even higher resolution when treating very small target volumes, for example, those encountered in SRS applications, a similar upsampling approach is currently under development for the OD 1600 SRS.

## CONFLICT OF INTEREST STATEMENT

Nicole Brand and Daniela Eulenstein are employees of PTW Freiburg GmbH. Elias Kempf and Jan Weidner are former employees of PTW Freiburg GmbH.
